# Structural investigation of human cystine/glutamate antiporter system x_c_^−^ (Sx_c_^−^) using homology modeling and molecular dynamics

**DOI:** 10.3389/fmolb.2022.1064199

**Published:** 2022-12-01

**Authors:** Tran Dieu Hang, Huynh Minh Hung, Pauline Beckers, Nathalie Desmet, Mohamed Lamrani, Ann Massie, Emmanuel Hermans, Kenno Vanommeslaeghe

**Affiliations:** ^1^ Department of Analytical Chemistry, Applied Chemometrics and Molecular Modelling FABI, Vrije Universiteit Brussel (VUB), Brussels, Belgium; ^2^ Institute of Neuroscience, Neuropharmacology Group, Université Catholique de Louvain, Brussels, Belgium; ^3^ Neuro-Aging and Viro-Immunotherapy Research Group NAVI, Vrije Universiteit Brussel (VUB), Brussels, Belgium

**Keywords:** cystine/glutamate antiporter, SLC7A11 (xCT), Sx_c_ -, molecular dynamics simulation, homology modeling, inhibitor, conformation

## Abstract

The cystine/glutamate antiporter system x_c_^−^ (Sx_c_^−^) belongs to the SLC7 family of plasma membrane transporters. It exports intracellular glutamate along the latter’s concentration gradient as a driving force for cellular uptake of cystine. Once imported, cystine is mainly used for the production of glutathione, a tripeptide thiol crucial in maintenance of redox homeostasis and protection of cells against oxidative stress. Overexpression of Sx_c_^−^ has been found in several cancer cells, where it is thought to counteract the increased oxidative stress. In addition, Sx_c_^−^ is important in the central nervous system, playing a complex role in regulating glutamatergic neurotransmission and glutamate toxicity. Accordingly, this transporter is considered a potential target for the treatment of cancer as well as neurodegenerative diseases. Till now, no specific inhibitors are available. We herein present four conformations of Sx_c_^−^ along its transport pathway, obtained using multi-template homology modeling and refined by means of Molecular Dynamics. Comparison with a very recently released cryo-EM structure revealed an excellent agreement with our inward-open conformation. Intriguingly, our models contain a structured N-terminal domain that is unresolved in the experimental structures and is thought to play a gating role in the transport mechanism of other SLC7 family members. In contrast to the inward-open model, there is no direct experimental counterpart for the other three conformations we obtained, although they are in fair agreement with the other stages of the transport mechanism seen in other SLC7 transporters. Therefore, our models open the prospect for targeting alternative Sx_c_^−^ conformations in structure-based drug design efforts.

## 1 Introduction

The cystine/glutamate antiporter system x_c_^−^ (Sx_c_^−^) is a plasma membrane transporter that is found in a wide variety of cells, including cells of the immune system, astrocytes and cancer cells (François Verrey et al., 2004; Cho and Shiro, 1990; S Bannai 1986; Sato et al., 1999; Hosoya et al., 2002; Piani and Fontana 1994). Accordingly, it plays a critical role in diverse parts of the body, especially in central nervous system (Fort et al., 2007; Hosoya et al., 2002; Eck and Wulf, 1989; S Bannai and Ishii 1988; S Bannai 1986; Dingledine et al., 1999; Christensen 1990). Sx_c_^−^ is a Na^+^-independent amino acid antiporter that regulates the cellular uptake of cystine in exchange for glutamate across the plasma membrane in a 1:1 ratio (Christensen 1990; S Bannai 1986; S Bannai and Kitamura 1980). As such, it plays an important role in maintaining the redox balance between extracellular cystine and cysteine (S Bannai and Ishii 1982; Anderson et al., 2007). In addition, its rate of cystine uptake regulates the biosynthesis of glutathione (GSH), a tripeptide thiol crucial for protection of cells against oxidative stress and toxicity of xenobiotic electrophiles, and a signal molecule entailed in cell cycle regulation, cellular proliferation and apoptosis (Miyamoto et al., 1989; Murphy et al., 1989; Murphy and Baraban 1990; Savolainen et al., 1998; Baker et al., 2002; Anderson et al., 2007; Lewerenz et al., 2013). Sx_c_^−^ is thus essential for the viability of both normal cells *in vitro* and malignant cells. Indeed, Sx_c_^−^ overexpression has been observed in several distinct cancer cells (Guo et al., 2011; LA et al., 2013; Yang and Douglas, 2014; Slosky et al., 2016; Ji et al., 2018) in association with an increased GSH production, boosting the cells’ antioxidant capacity and supporting their proliferation (Lo et al., 2008; Yang and Douglas, 2014; Koppula et al., 2018; Combs and DeNicola, 2019). Specifically, there is mounting evidence that short-term, specific inhibition of Sx_c_^−^ can lead to inhibited evolution and reduced drug resistance of a diversity of cancers without substantial side effects to the host (Lo et al., 2008). Moreover, the antiporter system is linked with multiple functions of the central nervous system and is important for the glutamate balance in many brain regions to ensure proper glutamatergic neurotransmission as well as to prevent excitotoxicity (Albrecht et al., 2010; R. Bridges et al., 2012b; de Bundel et al., 2011). Inhibiting Sx_c_^−^ has effects on emotional and cognitive aspects of behavior, drug addiction and neurological diseases like Alzheimer’s, Parkinson’s, Huntington’s, amyotrophic lateral sclerosis (ALS), multiple sclerosis and epilepsy (Massie et al., 2008; Lewerenz et al., 2013; Mesci et al., 2015; Fournier et al., 2017; Merckx et al., 2017). Pharmacological inhibitors of Sx_c_^−^ could thus be part of prospective treatments for cancer and various diseases. However, the currently known classes of Sx_c_^−^ inhibitors have unfavorable pharmacological properties including rapid metabolism and/or off-target effects (R. J. Bridges RichardJ. et al., 2012; Lewerenz et al., 2013). Therefore, discovering new molecules that selectively inhibit this antiporter would open the door to new treatments for the diseases mentioned above. In order to design novel small-molecule Sxc^−^ modulators, understanding the structure of Sxc^−^ is one of the most essential steps.

Sxc^−^ belongs to the heteromeric amino acid transporter (HAT) family wherein transporters consist of a heavy subunit (SLC3) and a light subunit (SLC7) joined *via* an extracellular disulfide bond (Bröer and Wagner 2002; Verrey et al., 2004; Palacín et al., 2005). The heavy chain of Sx_c_^−^, named 4F2hc, CD98 or SLC3A2, is found in multiple members of the SLC7 family of transporters and is responsible for the trafficking of the antiporter to the plasma membrane and stabilization of the light chain (Wagner, Lang, and Bröer 2001). It contains a single transmembrane helix with a glycosylated extracellular domain as C-terminus and an intracellular N-terminus. The light chain, also known as SLC7A11 or xCT, is a polytopic membrane protein. It determines the characteristics of the heterodimer - most importantly its substrate specificity. xCT comprises 12 transmembrane domains with both intracellular C- and N-termini which can undergo various conformational changes while switching the ligand accessibility between the intracellular and extracellular side.

At the time of our initial investigation, three homology models for Sxc^−^—or more specifically its light chain xCT - have been published, based on the crystal structure of bacterial transporters AdiC and ApcT (Deshpande, Sharma, and Bachhawat 2017; R. J. Bridges RichardJ. et al., 2012; Sharma and Anirudh 2019; Matti et al., 2013). However, because of the relatively low sequence homology to the template proteins, these models suffer from a number of shortcomings. Most importantly, transmembrane domains of the heavy chain as well as both N- and C-termini were omitted from the models. The latter was visible in the recently published cryo-EM structure of the human transporters LATs, namely LAT1/4F2hc, LAT2/4F2hc and b^0,+^AT/rBAT which have better sequence similarity with Sxc^−^ than AdiC and ApcT. The N-terminal helix in the light chain was resolved in the crystal structure of GkApcT (Jungnickel, Parker, and Newstead 2018). Several studies showed important roles of this region in the transport mechanism, conformational change and signaling of some receptors and proteins (Baillie, 2002; Kanki et al., 2003; Zhao et al., 2010; Kazmier et al., 2014; Millard et al., 2014; Martinac et al., 2017). In addition, a group of some close lysine residues in the N-terminal tail of LAT1 was recently reported to be critical for downregulation and PMA-induced ubiquitylation (Schwartz and Mchaourab 2022). Accordingly, we hereby present an effort to construct higher-quality structural models including both N- and C- termini in different conformations of the cystine/glutamate antiporter system, using multi-template homology modeling followed by refinement by means of Molecular Dynamics (MD).

At time of writing, the higher resolution Cryo-EM inward-open structure of Sx_c_-has recently been released (Parker et al., 2021). This structure was used to validate our inward-open homology model, showing excellent agreement. Conversely, experimental structures of Sx_c_-in other states are not yet available; we hope to fill this gap with our models for these conformations.

## 2 Computational methods

### 2.1 Homology models

The amino acid sequence of the human cystine/glutamate transporter xCT (SLC7A11) was retrieved from the protein database on the National Center for Biotechnology Information (NCBI) under the code NP_055146.1. For the building of homology models, proteins with at least 25% sequence identity (SI) were chosen with the ‘profile.build’ command of Modeller as well as on the NCBI using its run-BLAST (Basic Local Alignment Search Tool) functionality. Available 3D structures of homologous proteins are downloaded from the protein databank (PDB). At the time our initial investigation was carried out, bacterial transporters AdiC (PDB ID: 5J4I), MjApcT (PDB ID: 3GIA), the newly published structure of human transporters LAT1-4F2hc (PDB ID: 6IRT, 6IRS, 6JMQ) and bacterial cationic amino acid transporter GkApcT (PDB ID: 6F34, 5OQT) and bacterial transporter BasC (PDB ID: 6F2G) were identified as the most homologous models. After taking into account the sequence and structural similarity between these PDB entries as well as their crystallographic resolutions, the structures listed in Table 1 were retained as suitable templates. Using these templates, homology modeling was performed with Modeller 9.23, setting the parameters library_schedule = autosched.slow and md_level = refine.very_slow. Specifically, multi-template homology modeling runs were performed using two to five of the aforementioned suitable templates in several permutations. For each of these permutations, a preliminary test run was performed in which 10 models were generated, setting the maximum number of iterations to 5000. The quality of the models resulting from these preliminary test runs (data not shown) was critically evaluated based on the DOPE (Discrete Optimized Protein Energy) and GA341 scores from Modeller as well as agreement of the location of secondary and tertiary structure elements with respect to the available experimental data. This way, the most promising combinations of templates were selected for more exhaustive “production runs”, where the number of models was increased to 400 and the maximum number of iterations to 200000.

**TABLE 1:** Description of the studied templates.

PDB code	Name	SI (%)	Cr (Å)	Conformation	Protomeric composition	Year
6IRT	Lat1-4F2hc complex bound with BCH	48.3	3.50	Inward-open	Heterodimer	2019
6IRS	LAT1-4F2hc complex incubated with JPH203	48.3	3.30	Inward-open	Heterodimer	2019
6JMQ	LAT1-CD98hc complex bound to MEM-108 Fab	48.1	3.31	Inward-open	Heterodimer	2019
6F2G	Bacterial asc transporter crystal structure in open to in conformation	27.5	2.92	Inward-open	Monomer	2019
6F34	Bacterial cationic amino acid transporter (CAT) homologue bound to Arginine	26.4	3.13	Inward-occluded	Monomer	2018
3GIA	ApcT Transporter	28.0	2.32	Inward-facing	Monomer	2009
5J4I	l-arginine/agmatine antiporter	25.0	2.21	Outward-open	Homodimer	2016

### 2.2 Molecular dynamic simulations

Since the heavy chain 4F2hc plays a critical role in stabilizing the light chain in the membrane (Fotiadis, Kanai, and Palacín 2013; Rosell et al., 2014; Fort, Nicolàs-Aragó, and Palacín 2021), we added the transmembrane domain of 4F2hc to our homology models of xCT to ensure that our simulation system is stable and active in a membrane environment. Since no experimental structure of xCT was available at time of carrying out our study, this was accomplished by aligning the homology models with the available cryo-EM structure of LAT1-4F2hc, which possesses a very similar light chain structure and shares the same heavy chain. Following this alignment, the relevant part of 4F2hc could simply be copied into our models, manually adding a disulfide bond between Cys158 in the light chain xCT and Cys211 in 4F2hc.

CHARMM-GUI was used to embed full system x_c_^−^ (xCT-4F2hc) in a membrane, consisting of a mixed cholesterol, 1-palmitoyl-2-oleoyl-sn-glycero-3-phosphoethanolamine (POPE), and palmitoyl sphingomyelin (PSM) bilayer with a 1/1/1 ratio for both leaflets (Almeida et al., 2003; Jo et al., 2009; Wu et al., 2015). A rectangular cuboid box was used, with the membrane in the XY-plane. The lengths of the X- and *Y*-axis were initially set to 104 Å. The length of the *Z*-axis was equal to the thickness of the membrane plus a layer of water at both sides, the thickness of which was set to 22.5 Å. As system building option, the replacement method was applied. Ions were included in the water phase–specifically, it was chosen to consist of 0.15 M KCl.

Atomistic simulations with explicit solvent were performed with NAMD 2.14 (Phillips et al., 2005). All simulation parameters were left at their CHARMM-GUI defaults, which are summarized in the remainder of this paragraph. A time step of 2 fs was used with the SHAKE algorithm (Ryckaert, Ciccotti, and Berendsen 1977). The van der Waals interactions were smoothly switched off over 10 to 12 Å by a force-switching function (Steinbach and Brooks 1994); the long-range electrostatic interactions were calculated using the particle-mesh Ewald method (Essmann et al., 1998) with a mesh size of ∼1 Å and a sixth order spline interpolation. Langevin dynamics with a damping coefficient of 1 ps–1 was used to maintain a constant temperature of 303.15 K. Likewise, the pressure was kept at 1 bar using a Nosé-Hoover Langevin-piston barostat (Feller et al., 1998; Martyna, Tobias, and Klein 1998) with a piston period of 50 fs and a piston decay of 25 fs.

The solvent-accessible surface area (SASA) for our models and other proteins was calculated using VMD (Humphrey, Dalke, and Schulten 1996). Clustering was performed with the “gmx cluster” function implemented in GROMACS (Abraham et al., 2015) Specifically, the linkage criterion was used with an RMSD cut-off value between 1.0 and 1.2 Å. For each resulting cluster, the centroid was taken as a representative structure. The pore profile was calculated using the HOLE program (Smart et al., 1996).

### 2.3 Virtual screening and *in vitro* testing

Docking was carried out using AutoDock vina version 1.1.2 (Trott and Olson 2010) with modifications in its input/ouput routines for high-throughput screening of large libraries. In the present work, we used an adaptation of the 2-stage docking procedure described in (Pan et al., 2003; Yu and Mackerell 2017) against the Sx_c_-inward-open conformation. The initial library of drug candidates consisted of the subset of the ZINC database (Irwin et al., 2012) that was converted to AutoDock’s pdbqt format for use in the FightAIDS@Home (FAQ, 2020 “FightAIDS@Home. Https://Web.Archive.Org/Web/20211019203737/Http://Fightaidsathome.Scripps.Edu/- Archived Version from Http://Fightaidsathome.Scripps.Edu/” 2020) and Global Online Fight Against Malaria projects. This set of 6 132 751 compounds was initially docked with Vina’s “exhaustiveness” parameter set to four and subsequently reduced to 200 000 in the first stage of the protocol. In the second stage, the number of docking hits was further reduced to 1000 using a Vina exhaustiveness of 16. Out of those 1000 compounds, 11 were selected for *in vitro* testing based on structural diversity, drug-like properties and commercial availability, using DataWarrior version 5.5 (Sander et al., 2015). These 11 compounds were procured from Chembridge corp. (San Diego, CA, United States) and Vitas M Chemical ltd. (Causeway Bay, Hong Kong) through Molport, SIA (Riga, Latvia) and tested *in vitro* at a concentration of 10^–4^ M, following the protocol described in (Beckers et al., 2022).

### 2.4 System x_c_^−^ functional assay using [^3^H]-l-glutamate uptake

Glutamate uptake was performed as previously described by (Beckers et al., 2022). Briefly, transfected Chinese hamster ovary (CHO) cells expressing the human xCT were grown on 24-well plates. Culture medium was replaced with preheated Na^+^-free buffer for 20 min. After this incubation, the plate was placed on the surface of a 37°C water bath and the buffer was removed and immediately replaced with the same buffer supplemented with [^3^H]-l-glutamate at a concentration of 20 nM. When indicated, the tested compounds (number #1 to #11) or the pharmacological inhibitors homocysteic acid (HCA) and L-threo-3-hydroxyaspartic acid (LTHA) were added at a final concentration of 10^−4^ M. After 20 min incubation, the uptake was stopped by three rinses with ice-cold Na^+^-free buffer and cells were lysed with 0.1 M NaOH. A fraction of the lysate was collected and the radioactivity content was measured using the liquid scintillation solution Microsint 40 and the Topcount^®^ NXT Microplate scintillation and luminescence counter. Another fraction of the lysate was used for protein quantification using the Bradford method with the Bio-Rad protein assay. Results are expressed as pmol of radiolabelled glutamate transported per min per mg of protein.

## 3 Results and discussion

### 3.1 Homology models of the light chain xCT

In order to find the best set of templates to build multi-template homology models of xCT, permutations of multiple templates were evaluated. Some general observations are summarized here. The models based on all templates simultaneously showed a number of interruptions in the transmembrane helices that was deemed unlikely. The same shortcoming was also observed in single-template models using 3GIA as a template. Conversely, the single-template models based on the other templates did not feature excessive interruptions in the helices; in particular, the single-template models using 6IRT and 6IRS as a template appeared to be promising. Specifically, only the models based on 6IRT and 6IRS contained a lateral C-terminal helix in the region between Arg475 and Leu492, as this region was unresolved or absent in the other templates. While otherwise very similar, the models based on 6IRT had slightly longer transmembrane helices than those based on 6IRS. Meanwhile, a lateral helix consisting of N-terminal residues 26–39 was only observed in models built with 6F34.

In an attempt to conserve both the N-terminal and the C-terminal lateral helices, dual-template models were built from both 6IRT and 6F34. However, the resulting models again displayed excessive interruptions in the transmembrane helices; we speculate that this is due to the relative dissimilarity between the two templates. Luckily, during the process of systematically evaluating combinations of templates, it was found that adding 5J4I as a third template enhanced greatly the quality of the resulting models. This was attributed to the ability of 5J4I to reconcile the differences between 6IRT and 6F34; in particular, a number of important gaps in the sequence alignment were resolved by the inclusion of 5J4I.

Generalizing the above observations, “test runs” were performed using several permutations of templates (see “computational methods”), selecting suitable combinations based on the following criteria: 1) mostly uninterrupted transmembrane (TM) helices (with the obvious exception of the known interruptions in TM1 and TM6 that are thought to play a role in the transport mechanism) 2) containing lateral N- and C-terminal helices 3) displaying more secondary structure (i.e., a more “generic” indicator of quality in homology modeling).

#### 3.1.1 Models built from 6IRT, 5J4I and 6F34

In agreement with the results obtained in the preliminary triple-template homology modeling based on 6IRT, 5J4I and 6F34, the same templates were used in a more exhaustive “production run” generating 400 homology models (see “computational methods”). Overall, the resulting homology models are in line with a topological model for xCT based on mutagenesis/biotinylation experiments (Gasol et al., 2004). The analysis of the precise conformations in these models is discussed in the following paragraphs.

**FIGURE 1:**
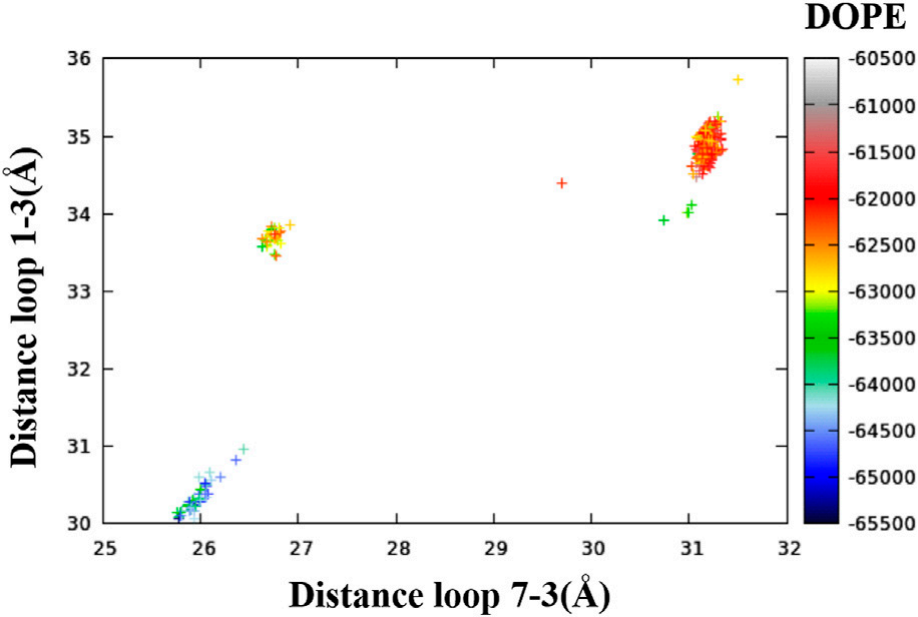
Relationship between the distances of loop one to three, the distance of loop seven to three and the DOPE score of the 400 conformers.

In order to map the conformational diversity among the 400 resulting models, the distances between the intracellular ends of several pairs of transmembrane helices were calculated. It was found that the distances between (the intracellular ends of) helices one and three and helices seven and three contained the most relevant information. In Figure 1, each of the 400 conformers is represented as a point in a plane defined by these two distances and colored by DOPE score (Modeller’s primary figure of merit, expressed as a free energy) (Eswar et al., 2006).^*^

Figure 1 readily shows three clusters of points, representing three distinct conformations based on the one to three and seven to three distances. For each cluster, in addition to the lowest DOPE point, we picked an additional five to six points in different regions of the cluster for visualization. The selection of the representative points given in Table 2 is based both on DOPE score and (manually assessed) structural characteristics. A similar analysis based on the distances between helices TM1 and TM3 and helices TM6 and TM3 is included in the Supporting Information (Supplementary Figure S1 and Supplementary Table S1).^47^

**TABLE 2:** Description of different selected models.

Cluster	Model	Distance loop 1–3 (Å)	Distance loop 7–3 (Å)	DOPE score	Structure
1	330	35.10	31.22	–63218.02	Wide open structure, shorter helix TM5
	286	35.09	31.21	–62557.37	Wide open structure, optimal alpha helices
2	127	33.57	26.64	–63548.59	Normal structure, no helix TM6b, shorter helix TM5
	189	33.73	26.72	–63208.85	Normal structure, no helix TM6b, optimal alpha helices
	283	34.122	26.77	–63361.71	Normal structure, optimal alpha helices
3	259	30.06	25.78	–65333.53	Close structure, no helix TM6b
	140	30.19	25.89	–64438.16	Close structure, optimal alpha helices
	136	30.97	26.43	–64037.43	Close structure, optimal alpha helices

##### 3.1.1.1 Inward-open conformation

**FIGURE 2:**
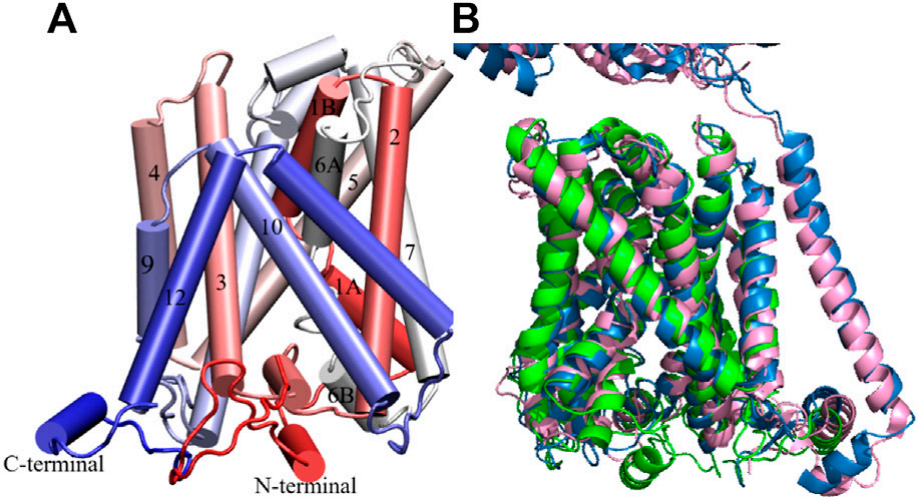
Homology model of xCT inward-open conformation. **(A)** Model 286. Helices are colored from red to blue from the N- to the C-terminus. **(B)** Superimposition of our homology model and low (Oda et al., 2020) and high resolution (Parker et al., 2021) Cryo-EM inward-open structures of Sx_c_. Homology model, low and high resolution Cryo-EM structures are colored in green, pink and blue, respectively.

Model **286** belonged to “cluster 1”, which features the largest intracellular distances between helices TM1 and TM3, between helices TM7 and TM3 and between helices TM6 and TM3 (*cf.* Table 2 and Supplementary Table S1) as well as a generally high proportion of alpha helices. Its conformation is in good agreement with LAT1 structure 6IRT, except for the position of the C-terminal helix (Supporting Information Supplementary Figure S2); on this basis, we classify it as an inward-open conformation. As shown in Figure 2A, the chosen xCT model possesses the 12 well-formed TM helices, positioned in a canonical LeuT fold. Both TM1 and TM6 of this model are disrupted by an unwound segment in the middle, with the names TM1A/TM1B and TM6A/TM6B correspond to the half helices, similar to other LeuT type-fold transporters. At time of writing, the higher resolution cryo-EM structure of Sx_c_-has been just released, it is thus used to validate our homology model. Figure 2B displays the superimposition of homology model **286** with the two recently published cryo-EM structures of Sx_c_-(Oda et al., 2020; Parker et al., 2021), showing an excellent agreement. Overall, the homology model (green) is even in better agreement with the very recent higher resolution structure by Parker *et al.* (Parker et al., 2021) (blue) than with the earlier structure by Oda *et al.* that was based on a consensus mutagenesis approach (Oda et al., 2020). Notable exceptions are the position of TM6B, which corresponds better to the Oda *et al.* structure, and the two terminal helices, in which N-terminal region is not resolved in either of the cryo-EM structures. In particular, the N-terminal lateral helix may play an important role in the transport mechanism of Sx_c_-, as has been observed in other members of the SLC7 family, where this domain is referred to as the intracellular gate (Zhao et al., 2010; Kazmier et al., 2014). In summary, even though neither of the Sx_c_-cryo-EM structures were available at the time our homology models were constructed, the models in “cluster one” show an excellent agreement with the experimental data. In addition, they exhibit secondary structure in the N-terminal domains, which is unresolved in the experiment and is thought to be important for the transport mechanism. Therefore, we considered these homology models as an optimal starting point for refinement by MD simulation.

##### 3.1.1.2 Inward-occluded model

**FIGURE 3:**
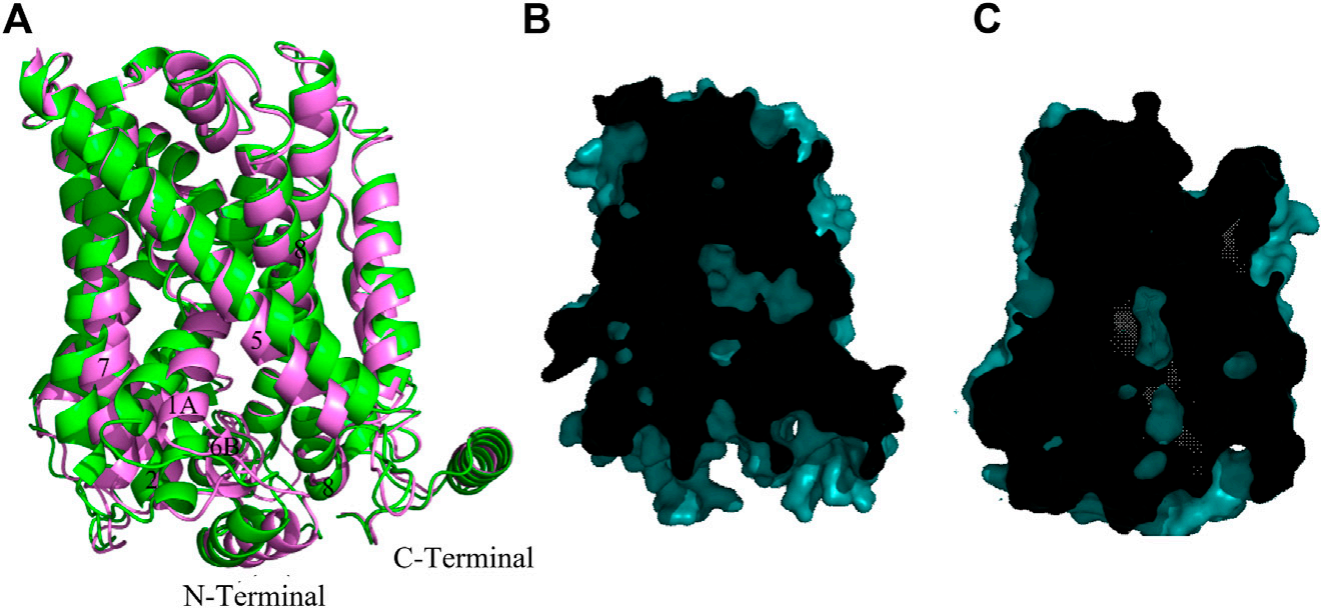
Homology model of xCT inward facing occluded conformation. **(A)** Superimposition of the model **286** (green) and model **283** (pink). **(B,C)** Sliced-surface representations of the model **283** and GkApcT inward-occluded structure (PBD: 6F34) (Jungnickel, Parker, and Newstead 2018) respectively.

In comparison with cluster one in Table 2, cluster two features a slight decrease in both the distance between TM1 and TM3 and between TM7 and TM3. Figure 3A shows the superimposition of model **286** (cluster 1) with the best model in cluster 2—model **283**. Compared with the inward-open model **286**, the cytoplasmic core domain of the light chain xCT in model **283** (formed by TMs 1, 2, 6, 7) moves towards the hash region (comprising TMs 3, 4, 8, 9), as demonstrated by the smaller distances from TM3 to the helices of the cytoplasmic core in Figure 1 and Supplementary Figure S1. These movements, along with major tilting of the cytoplasmic end of TM5 (Figure 3A), give rise to partial closing of the intracellular vestibule and result in tightly interacting TM1-TM5-TM8 segments, which is usually observed in transition from the inward-open to the inward-occluded conformation, as observed in previous studies (Errasti-Murugarren et al., 2019; Nicolàs-Aragó et al., 2021). Nevertheless, the intracellular side of the transporter is still noticeably more open than the extracellular side, which is tightly sealed through the interaction of TM1, TM6 with TM3, TM8. Similarity in sliced-surface representations between model **283** and the GkApcT inward-occluded structure (PBD: 6F34) (Jungnickel, Parker, and Newstead 2018), displayed in Figures 3B,C, also indicates our model **283** adopts an inward facing occluded state. Like model **286**, there exists a well-formed N-terminal helix in the structure of model 283, consistent with the GkApcT inward-occluded structure.

##### 3.1.1.3 Outward-occluded conformation

**FIGURE 4:**
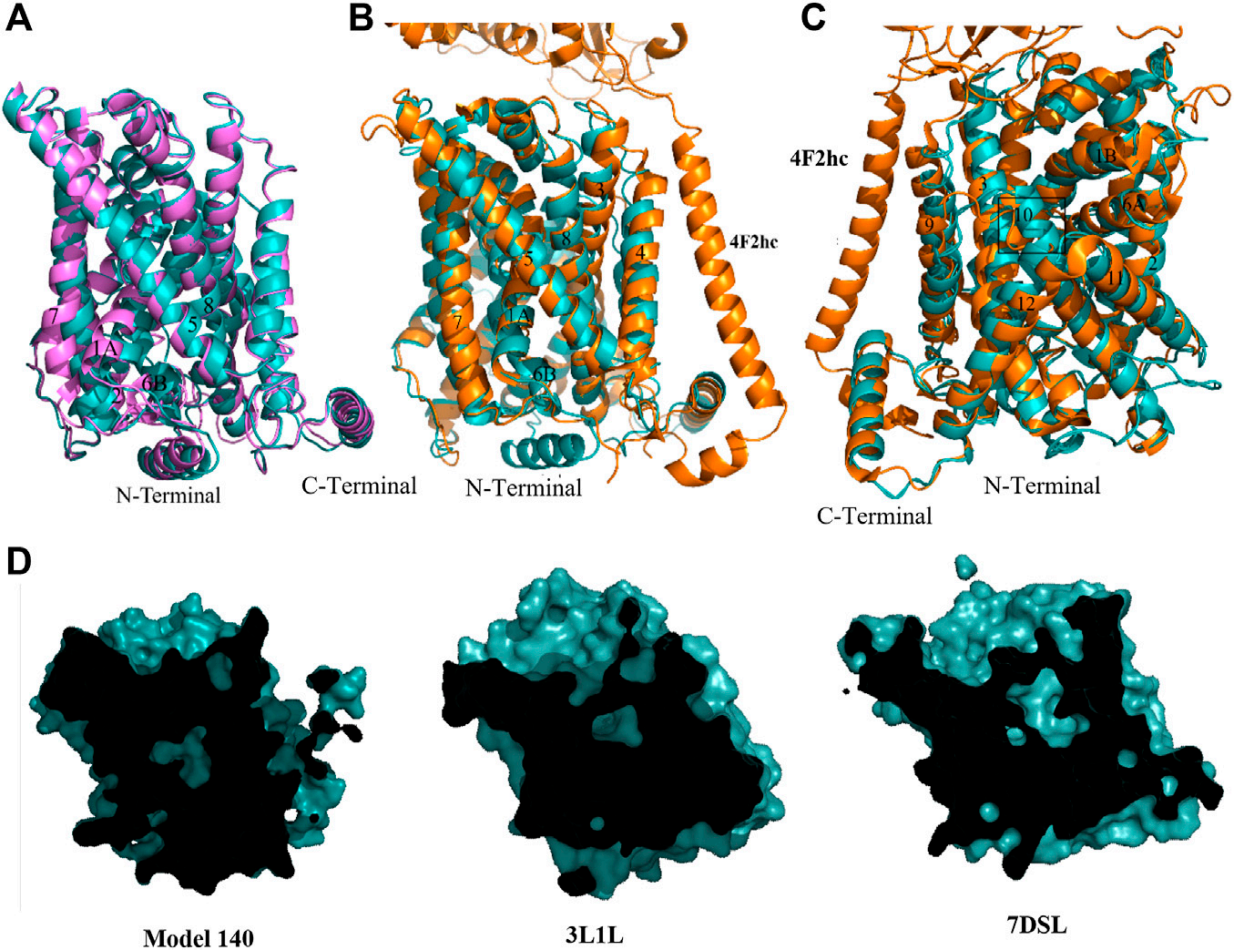
Homology model of xCT outward occluded conformation. **(A)** Overlay of model **283** (pink) and model **140** (cyan). **(B)** Overlay of model **140** (cyan) and Cryo-EM outward-occluded structures of human LAT1 with bound inhibitor JX-078 (PDB code: 7DSL, orange) from intracellular side. **(C)** Overlay of model **140** (cyan) and Cryo-EM outward-occluded structures of human LAT1 (7DSL) from extracellular side. **(D)** Sliced-surface representations of the model **140** and AdiC and human LAT1 outward facing occluded substrate-bound structure (3L1L and 7DSL, respectively).

Cluster 3 (Table 2) features the shortest intracellular distances between TM1 and TM3 and TM7 and TM3; as such, representative model **140** is considered a closed structure. Overall, the chosen model has the well-formed 12 TM helices and both N- and C-terminal helices that were also present in the other models. Alignment of model **140** with the inward facing occluded model **283** (Figure 4A) shows a major tilting of cytoplasmic ends of TM1A, TM6B and TM7, which appears to correspond to a closed intracellular gate. Indeed, structural superimposition of the human LAT1 outward inhibitor-bound occluded state (Yan et al., 2021) and model **140** shows the same positions of the helices at the intracellular side, especially TM1A, TM6B and TM7 (Figure 4B), indicating closure of the intracellular vestibule. Interestingly, the Cryo-EM outward-occluded structure of human LAT1 (which was published after conclusion of our homology modeling) features a previously unresolved loop preceding TM1, which is thought to contribute to the stabilization of the protein at the intracellular side upon hydrophobic interactions (Yan et al., 2021). The same loop occurs in our model, in a conformation that closely resembles the Cryo-EM structure (Figure 4B). Furthermore, at the extracellular side, the model features identical positions of TM1B and TM6A. Conversely, the positions of the extracellular ends of TM10 and TM9 in our model differ from those in the LAT1 outward-occluded structure (Figure 4C). This can be understood as follows: interaction between the hydrophobic tail of the inhibitor JX-078 bound in the LAT1 structure with some residues, including Ph252 on TM6, and VAL396 and Ile397 on TM10 led to pushing TM10 away and disorder of extracellular end of TM10 (Essmann et al., 1998). In addition, a similarity in cross-sectional surface representations is observed when comparing our model with both AdiC and human LAT1 outward facing occluded substrate-bound structure (Figure 4D). Given these observations, we will assume that model **140** exhibits an outward-occluded conformation.

#### 3.1.2 (Outward-open) model based on 6F34, 5J4I and 7DSL

**FIGURE 5:**
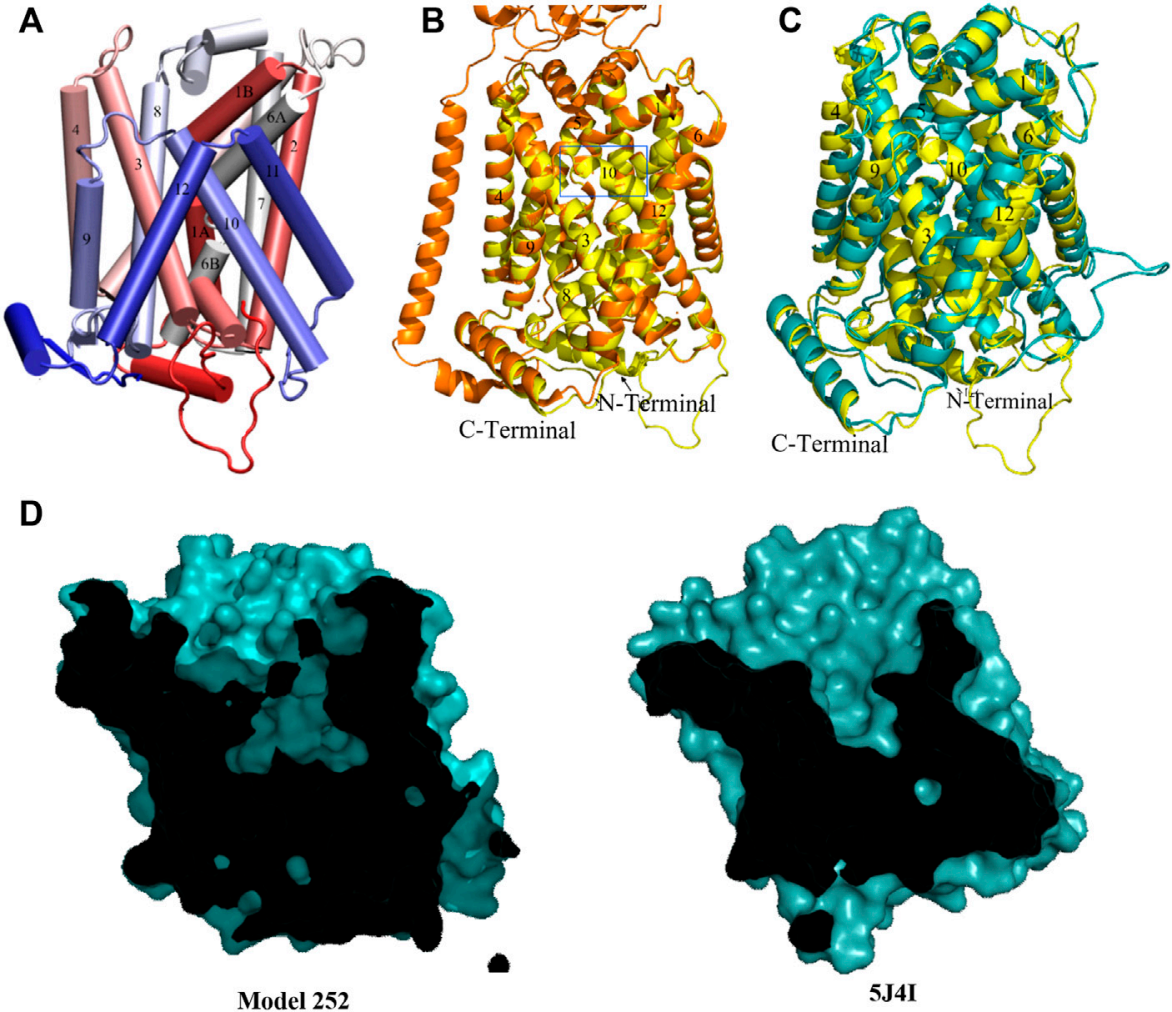
**(A)** Homology model of model 252. **(B)** Overlay of model **252** (yellow) and Cryo-EM outward-occluded structures of human LAT1 with bound inhibitor JX-078 (PDB code: 7DSL, orange) from intracellular side from extracellular side. The most different region (TM10) between two structures remarked in the blue box. **(C)** Overlay of model **252** (yellow) and model **140** (cyan). **(D)** Sliced-surface representations of the model **252** and AdiC outward-open substrate-free structure (5J4I).

Upon the release of the outward-occluded inhibitor-bound cryo-EM structure of human LAT1 (2021, PDB code:7DSL) (Yan et al., 2021), another homology modeling run was performed using 6IRT, 5J4I and 7DSL (instead of 6F34) as templates. This time, after analyzing the 400 models, only one conformation was found. The structure, named model **252** (Figure 5A) has a high proportion of well-formed structure elements (both alpha helices and beta sheets) and a correspondingly high DOPE score. The model is in fair agreement with the LAT1 template 7DSL even in the N-terminal loop; however, the loop of TM10 in the template turns out to be helical in our model (Figure 5B). Relative to outward-occluded model **140**, model **252** exists in a more open conformation in extracellular side (Figure 5C), especially when considering helices TM4, TM9 and TM10, out of which the latter is thought to play an important role in the transition between the outward-open and the outward-occluded state (Gao et al., 2010; Yan et al., 2021). A crossed-section surface of model **252** shows the central cavity harboring the substrate binding site opened to the extracellular space but without access to the cytoplasm. This cavity is also larger than in model **140** and in the LAT1 outward-occluded template (Figure 4D and Figure 5D) and similar to that of AdiC outward-open substrate-free state (5J4I). This is clearly seen in Table S2, which contains the calculated SASA for a selection of residues in the regions of TM1, 6, 3, 8 and 10 that form the binding pocket. Accordingly, we speculate that model **252** is representative of the outward-open state of xCT.

### 3.2 Models of Sx_c_^−^ refined by means of MD simulation

#### 3.2.1 Inward-open conformation

**FIGURE 6:**
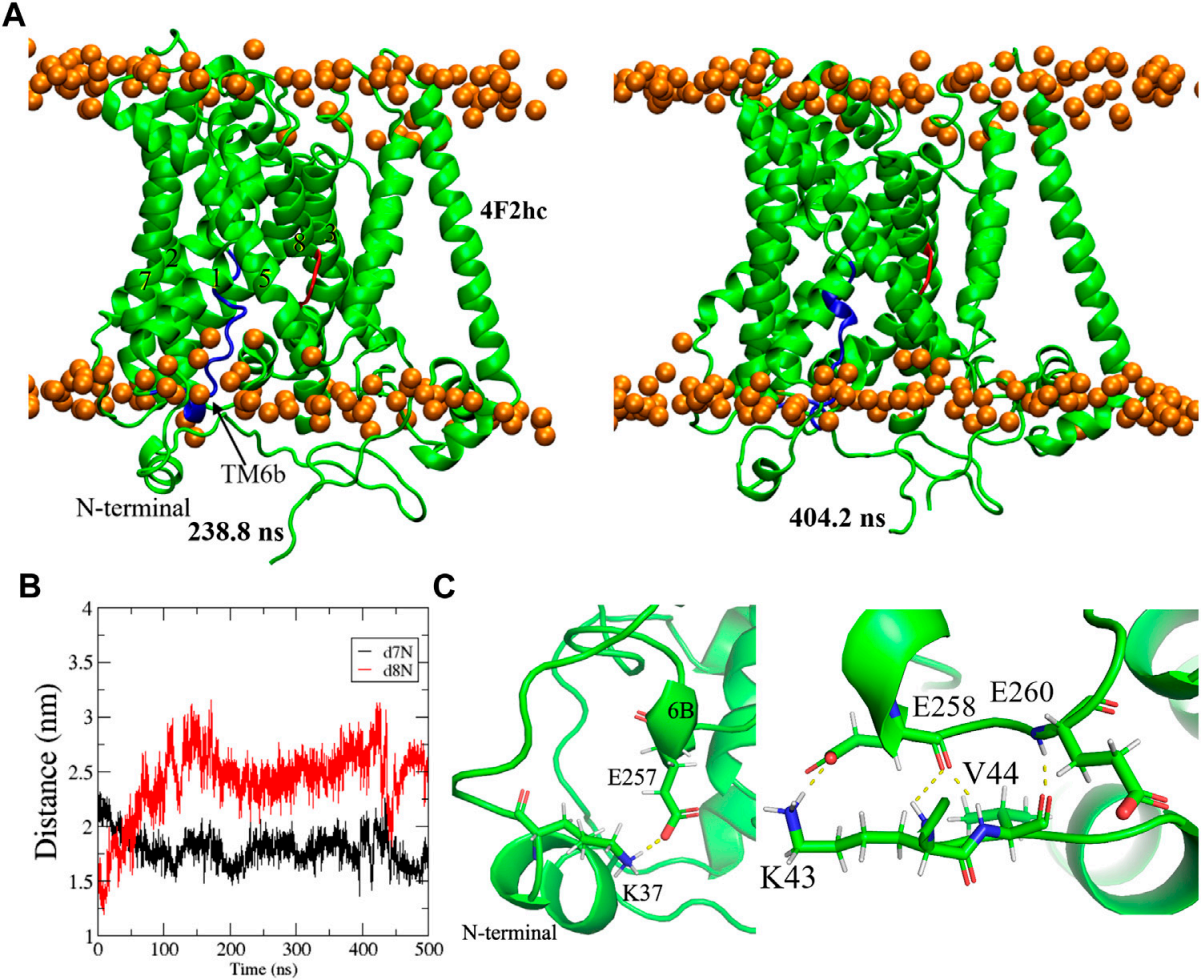
**(A)** Representative snapshots of equilibrium inward-open Sx_c_^−^ structure in membrane at different times of simulation. TM6b and a turn of TM8 are labeled by the blue and red, respectively. The intracellular surface is at the bottom. **(B)** Distance from the N-terminal helix to the intracellular loops between TM8 and TM7 during the MD simulation. **(C)** A close-up view of the interaction of TM6B and N-terminal helix.

The representative inward-open homology model (**286**) was taken as a starting point for a 500 ns MD simulation. As is apparent from the Root-Mean-Square-Distance (RMSD) of the backbone atoms (Supplementary Figure S3A), the structure is fully stabilized at 220 ns. Figure 6A shows two representative equilibrated conformations, obtained from clustering regularly spaced snapshots by backbone RMSD at 238.8 ns and 404.2 ns, respectively. Molprobity (Williams et al., 2018) was used to validate our MD refined model. For this purpose, inward-open representative conformation at 238.8 ns was minimized for 500 steps in order to relax close contacts and imperfect bond lengths and angles. Indeed, such “artificial” imperfections are inevitably present in (thermalized) MD snapshots and are inconsequential for the purpose of docking but would be penalized by Molprobity. The resulting Molprobity score was 0.882, where structures scoring below two are expected and scores near one are enviable as high-quality structures (Feig 2016). In addition, the RMSD of all C alpha atoms between the aforementioned representative conformation and the experimental structure (Parker et al., 2021) was 2.4 Å, indicating fair agreement. Some differences were observed between the original model and the equilibrated structures. Specifically, comparing the structures in Figure 2 and Figure 6A reveals a significant shift in the N-terminal helix from the middle of the intracellular vestibule to the bundle domain of the xCT formed by TMs 1, 2, 6, and 7. This can be clearly seen through a big change in distances between (the intracellular ends of) TM8 and N-terminal helices (referred to TM8-N) and between TM7 and N-terminal helices (TM7-N), given in Figure 6B. Indeed, the movement of the N-terminal domain induces a decrease of 5 Å in the TM7-N distance while TM8-N distance increases from 15 Å to 26 Å, thus leading to opening of the protein toward the cytoplasm. The Root-Mean-Square fluctuation (RMSF) for all residues of the light chain was also calculated in order to quantify to what extent the different domains of the protein fluctuate during the entire 500 ns simulation (Supplementary Figure S3B). Clearly, the N-terminal domain is highly mobile, in agreement with the fact that it is not resolved in any of the experimental inward-open APC transporter structures.

Interestingly, TM6B (given as the blue helix in Figure 6A) to some extent lost its highly regular alpha helical structure from the homology modeling, instead adopting a somewhat less regular alpha-like helical structure that later turned out to be in agreement with the higher resolution cryo-EM structures (Parker et al., 2021). Intriguingly, the authors of those structures (Gasol et al., 2004) reported an increased stability of this helix in the presence of cystine, concluding that this structural transition may play an important role in intracellular gating during transport, in agreement with substrate accessibility investigations (Gasol et al., 2004). The appearance of this unwound region of TM6B in our models may be a consequence of including the N-terminal helix region. Indeed, Figure 6C shows several interactions between TM6B and this N-terminal region.

It is also noteworthy that our xCT structure obtained after MD simulation possesses the same unwound region between GLY333 and Phe336 of TM8 (the red part in Figure 6A) as the experimental apo structure (Parker et al., 2021). This was deemed as an important finding for the transport mechanism of xCT (Parker et al., 2021).

Finally, we wish to point out that the MD structure generally offers an improvement upon the homology model by including the heavy chain 4F2hc. The positioning of this heavy chain (Supplementary Figure S4A) and its interactions with the light chain xCT are perfectly in line with the Cryo-EM Sx_c_^−^ structure (Parker et al., 2021). Specifically, the transmembrane segment of 4F2hc engages in extensive hydrophobic interactions with TM4 of xCT (Supplementary Figure S4C), which is also seen in LAT1, with supplemental interactions with the C-terminal lateral helix of the light chain (Supplementary Figure S4D), as confirmed by the Cryo-EM structure.

#### 3.2.2 Inward facing occluded conformation

**FIGURE 7:**
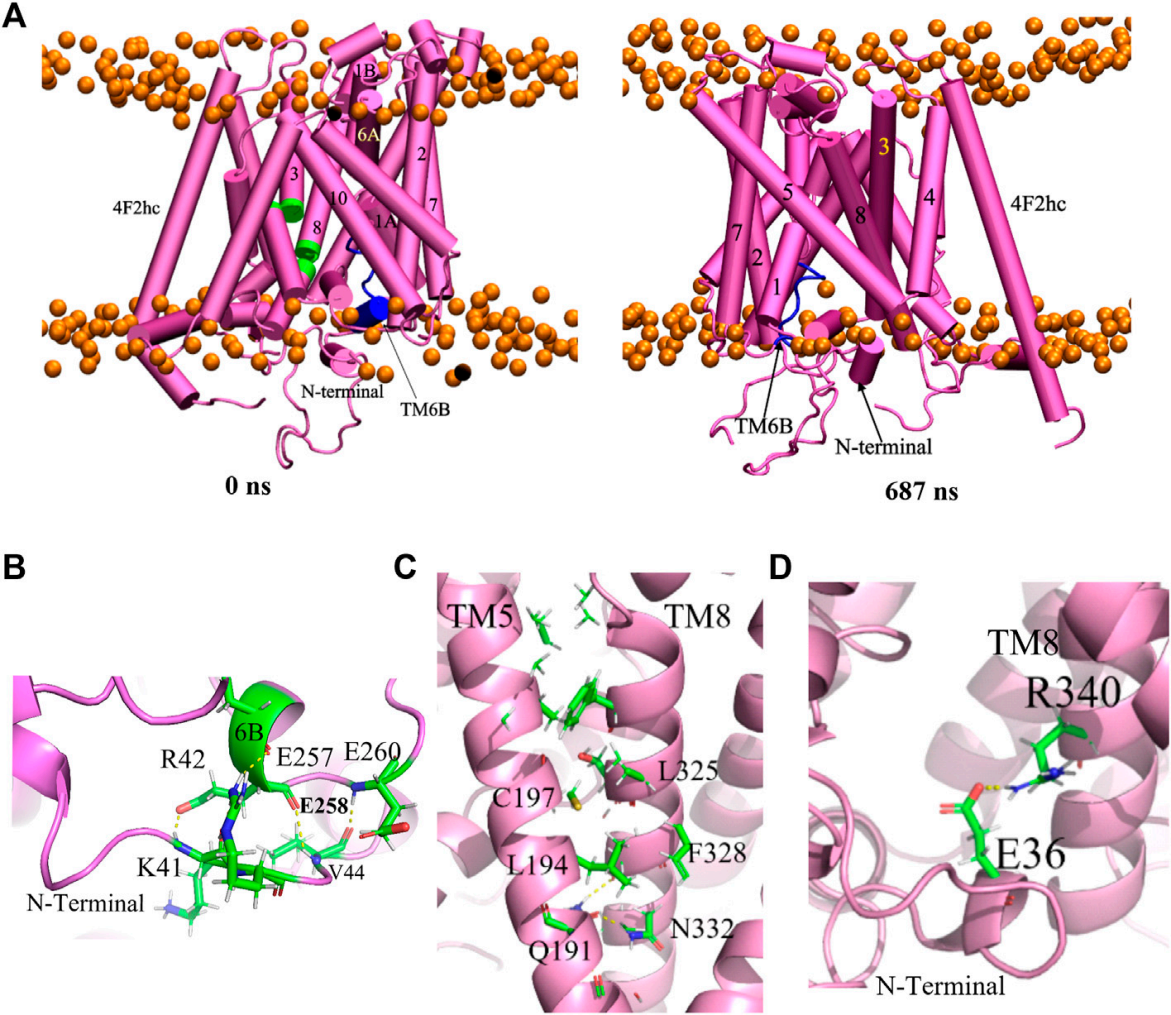
**(A)** Reference structure at 0 ns and representative equilibrium conformation at 687 ns of inward-occluded Sx_c_^−^ structure in membrane. TM6B and loops of TM3 and TM8 are labelled by the blue and green, respectively. The intracellular surface is at the bottom. A close-up view of **(B)** the interaction of TM6 and N-terminal region **(C)** the interaction of TM5 and TM8 and **(D)** N-terminal helix with TM8.

Similarly as in the previous subsection, the inward-occluded model (model **283**) was subjected to a 1000 ns MD simulation. The backbone RMSD indicates that the model reaches equilibrium rapidly after 90 ns and the system remains stable during the remainder of the simulation (Supplementary Figure S5A). Figure 7A shows the starting structure along with a representative conformation at 687 ns Interestingly, local interruptions in the alpha helical structures of TM3 and TM8 in the original homology model (green parts in Figure 7A) spontaneously disappeared during the simulation. The overall conformation remained inward-occluded, with only a minor shift in the position of the N-terminal helix compared to the homology model. This is also reflected in the per-residue RMS fluctuations, which are highest in the latter region (Supplementary Figure S5B).

Like in the inward-open structure, the TM6B alpha helix became somewhat distorted in this inward-occluded conformation. Interactions of TM6B with the N-terminal region, which (as discussed above) may play a role in modulating the conformation of TM6B, are shown in Figure 7B. This would appear to be a unique feature of the xCT transporter that is absent in other SLC7 structures, to the best of our knowledge (Parker et al., 2021). Furthermore, in contrast to the apo inward-open structure, the unwound region of TM8 is rigid helical in the present inward-occluded conformation. This can be explained by interactions between TM5 and TM8 (Figure 7C), which were not observed in the apo inward-open structure (Parker et al., 2021). Indeed, transition from the inward-open to the inward-occluded conformation involves a relatively large movement of TM1A to the hash domain formed by TM3, 4, 8, 9, resulting in TM1-TM5-TM8 coordination. In addition, the N-terminal helix appears to stabilize the formerly unwound region of TM8 *via* a salt bridge between Glu36 and Arg340 (Figure 7D).

#### 3.2.3 Outward-occluded conformation

**FIGURE 8:**
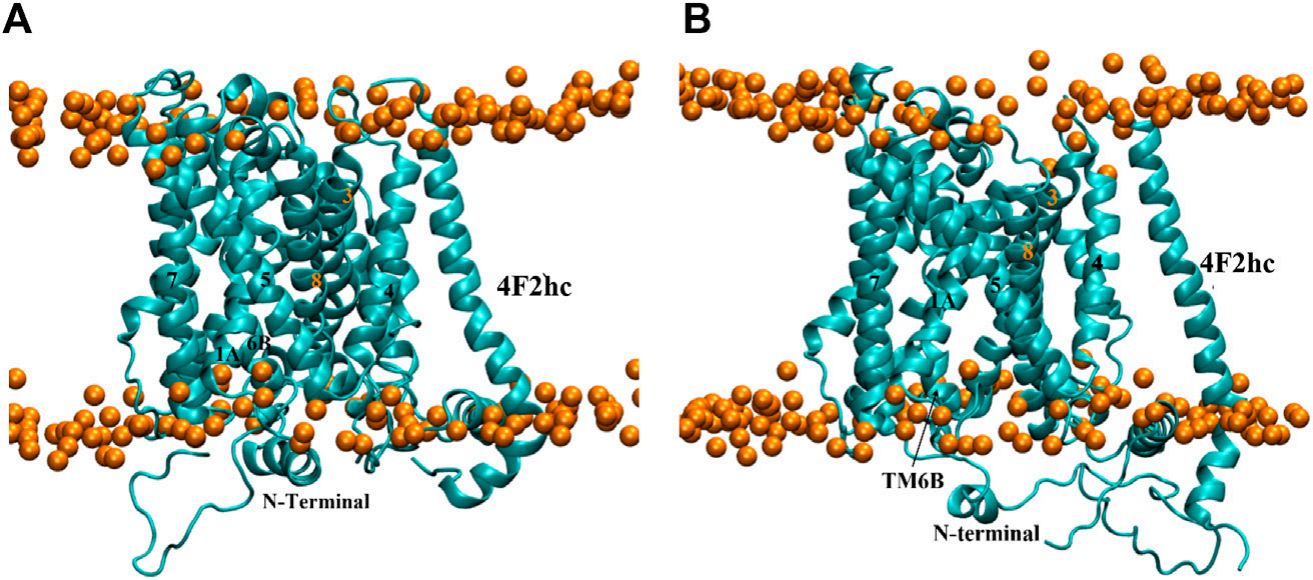
**(A)** Reference structure of model **140** at 0 ns and **(B)** Representative equilibrium conformation at 272.6 ns in membrane. The intracellular surface is at the bottom.

As with the other homology modeled conformations, a 500 ns MD simulation was started from model **140**. The RMSD time series in Supplementary Figure S6 illustrated that the model is fully stabilized at 140 ns Interestingly, a substantial conformational change happened in this time span. Contrary to the outward-occluded starting structure (Figure 8A), the equilibrium conformation has the same structure as the inward-open model (Figures 8B, 6A) with both TM1A and TM6B tilted sharply away from the hash domain, opening the intracellular side. This suggests that the ligand-free outward-occluded conformation of xCT is not stable, consistent with the observation that all currently available experimental outward-occluded structures of APC family members are substrate- or inhibitor-bound. It thus suggests that the outward-occluded conformation might function as a substrate-specific checkpoint (Beckstein and Naughton 2019; Drew et al., 2021), i.e., a state that cannot be traversed in the absence of a ligand, precluding the transporter from leaking non-substrate molecules, ions and solvent.

#### 3.2.4 Outward-open conformation

**FIGURE 9:**
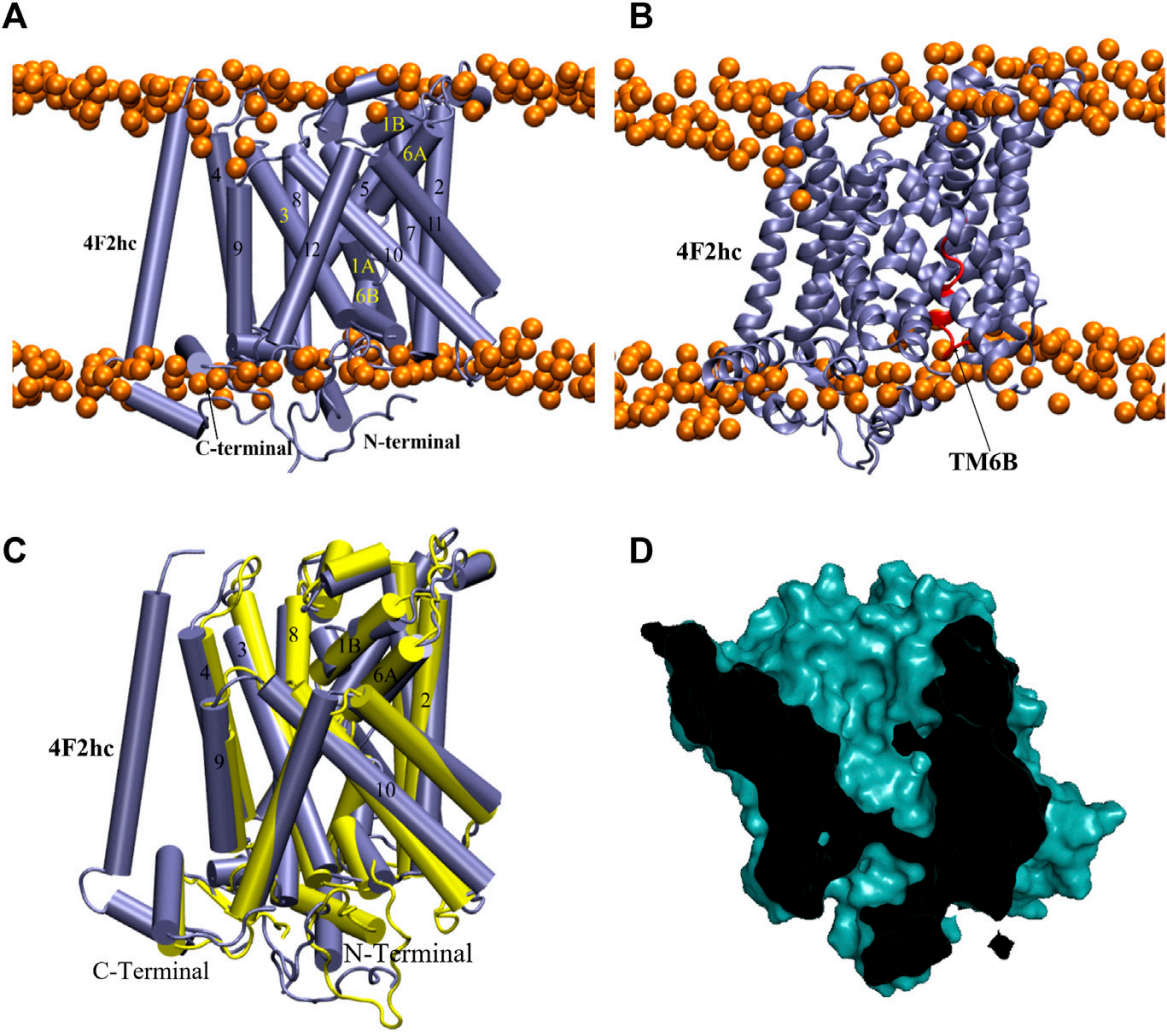
MD simulation structure of outward-open model. **(A,B)** Representative equilibrium snapshot of model **252** at 226.0 ns in membrane. The intracellular surface is at the bottom. **(C)** Structural overlay of model **252** before and after MD simulation (yellow and ice-blue, respectively). **(D)** Sliced surface representation at 226.0 ns.

For the outward-open conformation, a 600 ns MS simulation was run on model **252**, resulting in the RMSD time series in Supplementary Figure S7. The latter indicates that the structure reached an equilibrium after only a few nanoseconds and remained stable during the rest of the trajectory. A representative structure at 226.0 ns is shown in Figure 9A, B. Like the other models, TM6B adopted an imperfect alpha helix-like structure during the simulation (Figure 9B). Its interaction (Glu258-Glu260) with the N-terminal region (Val44) is shown in Supplementary Figure S8. Structural alignment of the representative structure with the starting conformation revealed major movements in TM1, TM6, TM3 and TM10 (Figure 9C), of which TM10 undergoes the most pronounced tilt. However, contrary to the outward-occluded conformation, the conformation remained outward-open. Indeed, the central cavity harboring the substrate binding site has opened even more to the extracellular side, as shown in the sliced surface representation (Figure 9D) and corroborated by the SASA value of the binding site (Supplementary Table S2). Notably, when focusing solely on the shape of the binding cavity, the representative conformation from MD (Figure 9D) resembles the outward-open state of AdiC (Figure 5D) more than the original homology model did (Figure 5C).

Similar to the inward-occluded conformation, the N-terminal helix is located in front of intracellular gate. This further supports the idea that a salt bridge between the N-terminal domain and TM8 might plays a role in stabilizing the continuous alpha helical conformation of TM8 and the closed state of the intracellular channel.

#### 3.2.5 Comparison of three distinct conformations

**FIGURE 10:**
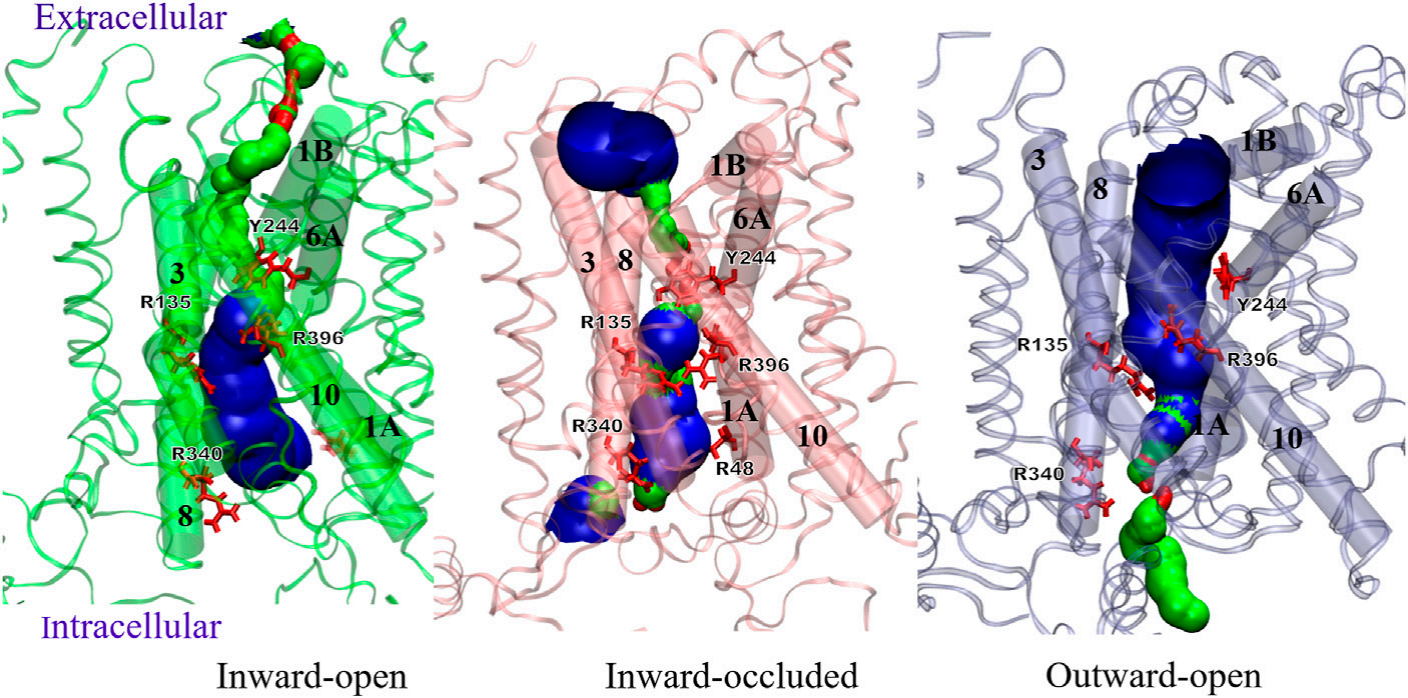
Comparison of Sxc-pore profiles in different conformations using the HOLE program.

The conformational changes of Sxc-can be clearly seen from Figure 10, showing the pore lining residues and helices of three distinct states, namely inward-open, inward-occluded and outward conformation. The transition from inward-open to inward occluded indicates the major movement of TM1A towards the hash region, facilitating the mentioned interaction of TM1-TM5-TM8 and inducing the smaller pore and vestibule at the intracellular side. Conversely, the outward-open state has the widest pore at the extracellular side and the smallest pore at the intracellular side among the three models. Supplementary Figure S9 shows the differences in the geometries of residues Arg396 on TM10 that undergoes the most pronounced tilt in the outward-open states and Tyr244 on TM6B between the inward-open and outward-open conformation. Different from the outward-open conformation (orange), the position of both residues in the inward-open state (red) tends to seal the cytoplasmic gate. This observation is in line with the hypothesis of (Parker et al., 2021), that defined Arg396 as an essential factor of specificity for transport mechanism of Sxc-.

### 3.3 Virtual screening and preliminary *in vitro* testing

**FIGURE 11:**
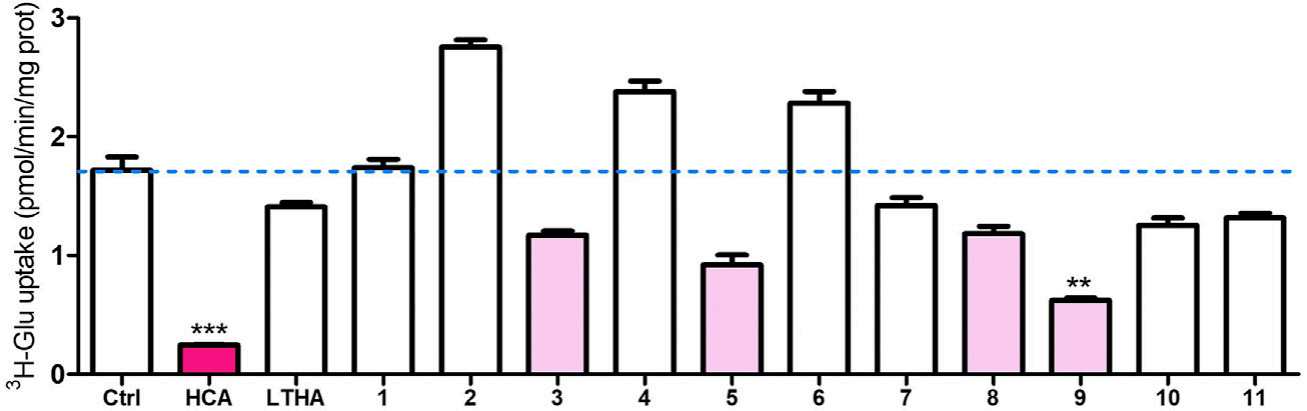
Preliminary results of ^3^H-Glutamate uptake assay at an inhibitor concentration of 10^–4^ M. Ctrl: control; HCA: homocysteic acid (known system x_c_^−^ inhibitor); LTHA (inhibitor of the excitatory amino acid transporters): L-threo-3-hydroxyaspartic acid. 1–11: virtual screening hits (arbitrary numbering). Data shown are the mean with SEM of the uptake capacity from three independent experiments; **p < 0.01, ***p < 0.001.

Docking was performed against our inward-open model **m286** as summarized in § 2.3. In a preliminary series of *in vitro* tests at a single concentration, the resulting 11 compounds were subjected to the ^3^H-glutamate uptake assay described in (Beckers et al., 2022). As shown in Figure 11, one of the compounds (“compound 9”) displayed a modest but statistically significant inhibitory activity. In addition, a small increase in apparent Sx_c_^−^-mediated transport was observed for three of the compounds (compounds 2, and 6). As this effect did not reach statistical significance in the present set of preliminary results, we will refrain from speculating on its meaning. Nevertheless, the fact that some degree of Sx_c_^−^ modulation was observed experimentally supports the validity of the inward-open model. This opens the prospect of finding Sx_c_^−^ inhibitors by screening against other conformations, some of which might be more druggable. A more elaborate pharmacological report on the subject of our virtual screening and testing efforts is in preparation.

## 4 Conclusion

Despite being the result of homology modeling followed by MD, our inward-open state shows an excellent agreement with the recently published cryo-EM structure. Interestingly, it contains an N-terminal alpha helix that was not resolved in any experimental structure because of its high mobility. Our models of the other states hint that this domain may play a role in the gating mechanisms. Indeed, the same workflow that gave rise to the aforementioned inward-open state also yielded inward-occluded and outward-open conformations that were stable during more than 250 ns of MD and closely resembled corresponding states of other transporters in the SLC7 family. Conversely, while the homology modeling also yielded a putative outward-occluded state, this state spontaneously converted to the inward-open state during the MD simulation. This is in line with the fact that experimental outward-occluded states of SLC7 family members always include either a substrate or an inhibitor, and suggests that the outward-occluded conformation might function as a substrate-specific checkpoint that prevents the transporter from leaking non-substrate molecules, ions and solvent.

## Data Availability

The original contributions presented in the study are included in the article/Supplementary Materials, further inquiries can be directed to the corresponding authors.
