# Supplementary material for: Structural investigation of human cystine/glutamate antiporter system xc − (Sxc −) using homology modeling and molecular dynamics
Source: Front Mol Biosci. 2022 Dec 1;9:1064199. doi: 10.3389/fmolb.2022.1064199 (PMC9751330; doi:10.3389/fmolb.2022.1064199)
Supplement: Supplementary file 1 [file DataSheet1.pdf]

Supplementary Material

1 Tables

Cluster	Model	Distance loop 1-3 (Å)	Distance loop 6-3 (Å)	DOPE score	Structure
**1**	**330**	35.10	25.41	-63218.02	Wide open structure, shorter helix TM5
	**286**	35.09	25.51	-62557.37	Wide open structure, optimal α-helices
**2**	**127**	33.57	23.71	-63548.59	Normal structure, no helix TM6b, shorter helix TM5
	**189**	33.73	23.87	-63208.85	Normal structure, no helix TM6b, optimal α-helices
	**283**	34.122	24.07	-63361.71	Normal structure, optimal α-helices
**3**	**259**	30.06	20.76	-65333.53	Close structure, no helix TM6b
	**140**	30.19	20.85	-64438.16	Close structure, optimal α-helices
	**136**	30.97	22.15	-64037.43	Close structure, optimal α-helices

Table S1. Description of different selected models

Model	**Model 140**	**Model 252**	**Equilibrium Model 252**
SASA	2471	2968	3089

Table S2. The calculated solvent-accessible surface area (SASA, $Å^2$) of some main residues in the regions of TM1 (residues 54–72), TM6 (residues 233–253), TM3 (residues 130–153), TM8 (residues 317–341) and TM10 (residues 385–406) wrapping the binding pocket of our models.

2 Figures

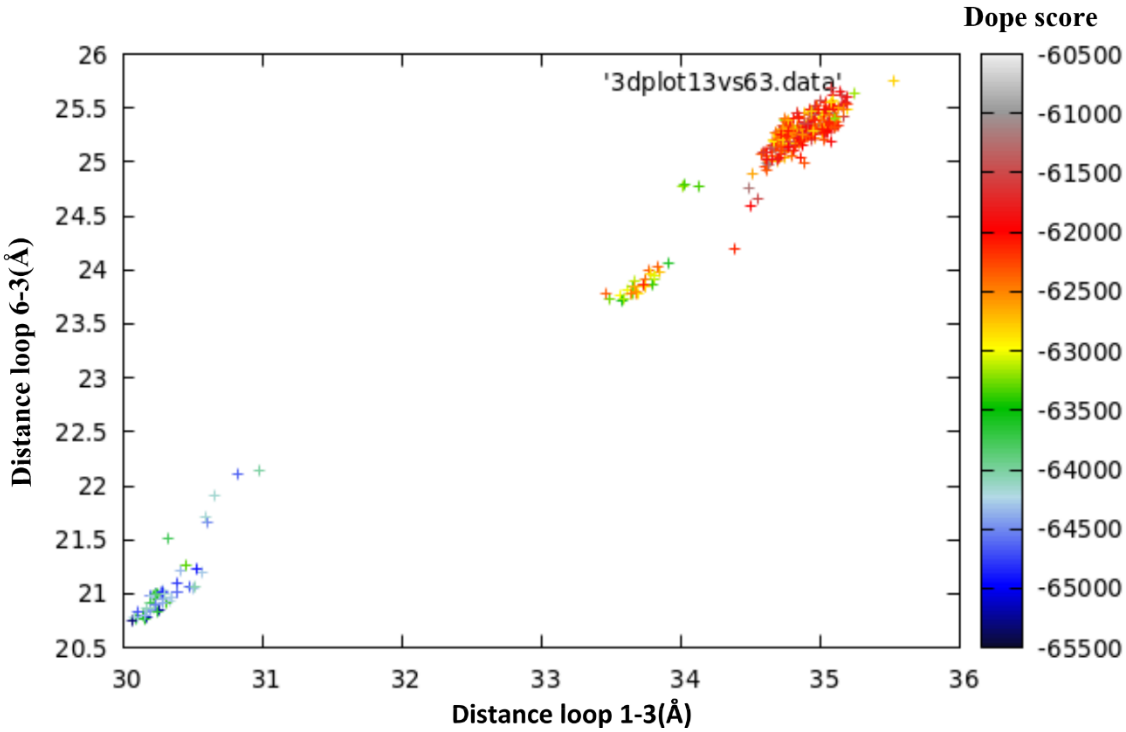

Figure S1. relationship between the distances of loop 1-3, the distance of loop 6-3 and the DOPE score of the 400 conformers using 6IRT, 6F34 and 5J4I as templates.

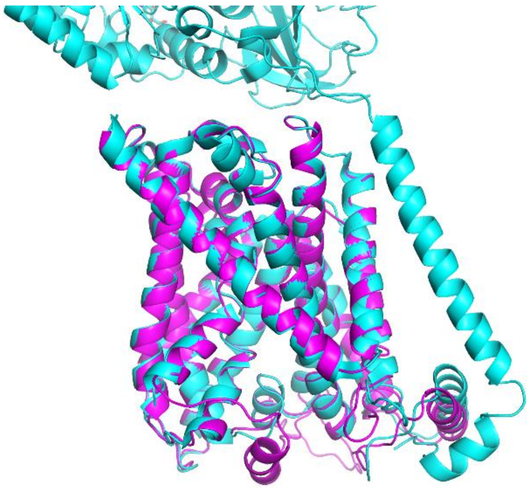

Figure S2. Superimposition of our homology model and low [46] and Cryo-EM inward open structures of LAT1 (PDB: 6IRT). The homology model and Cryo-EM structures are colored in pink, grey and cyan, respectively.

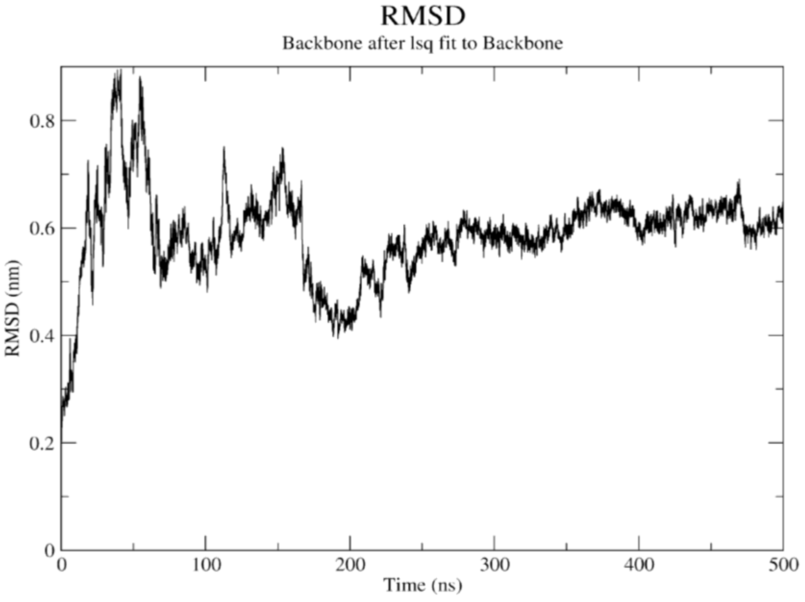

(A)

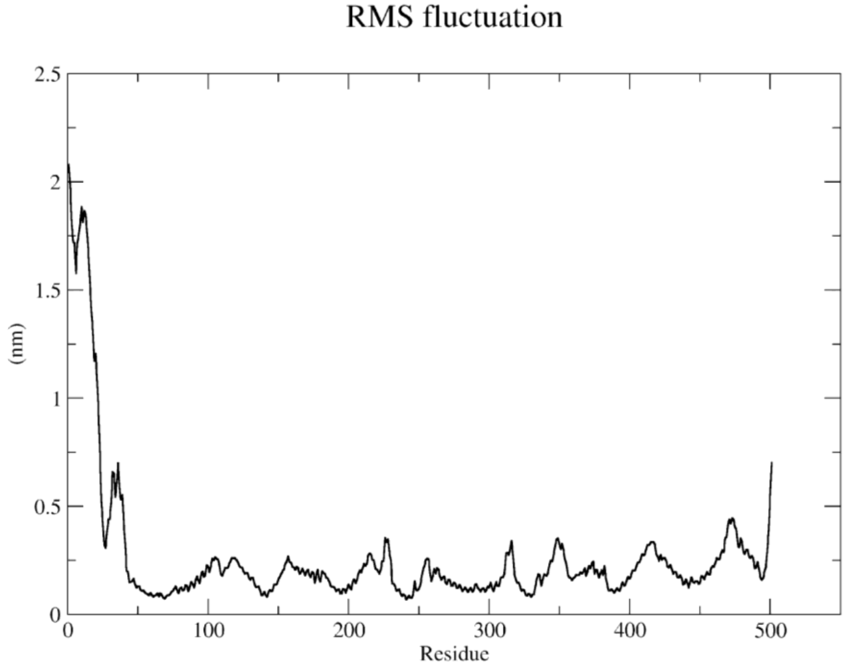

(B)

Figure S3. MD simulation analysis of inward open conformation of xCT. **(A)** Backbone RMSD (in nm) of model **286** during 500 ns. **(B)** Root-Mean-Square fluctuation (RMSF) for all residues of the light chain xCT during 500 ns simulation.

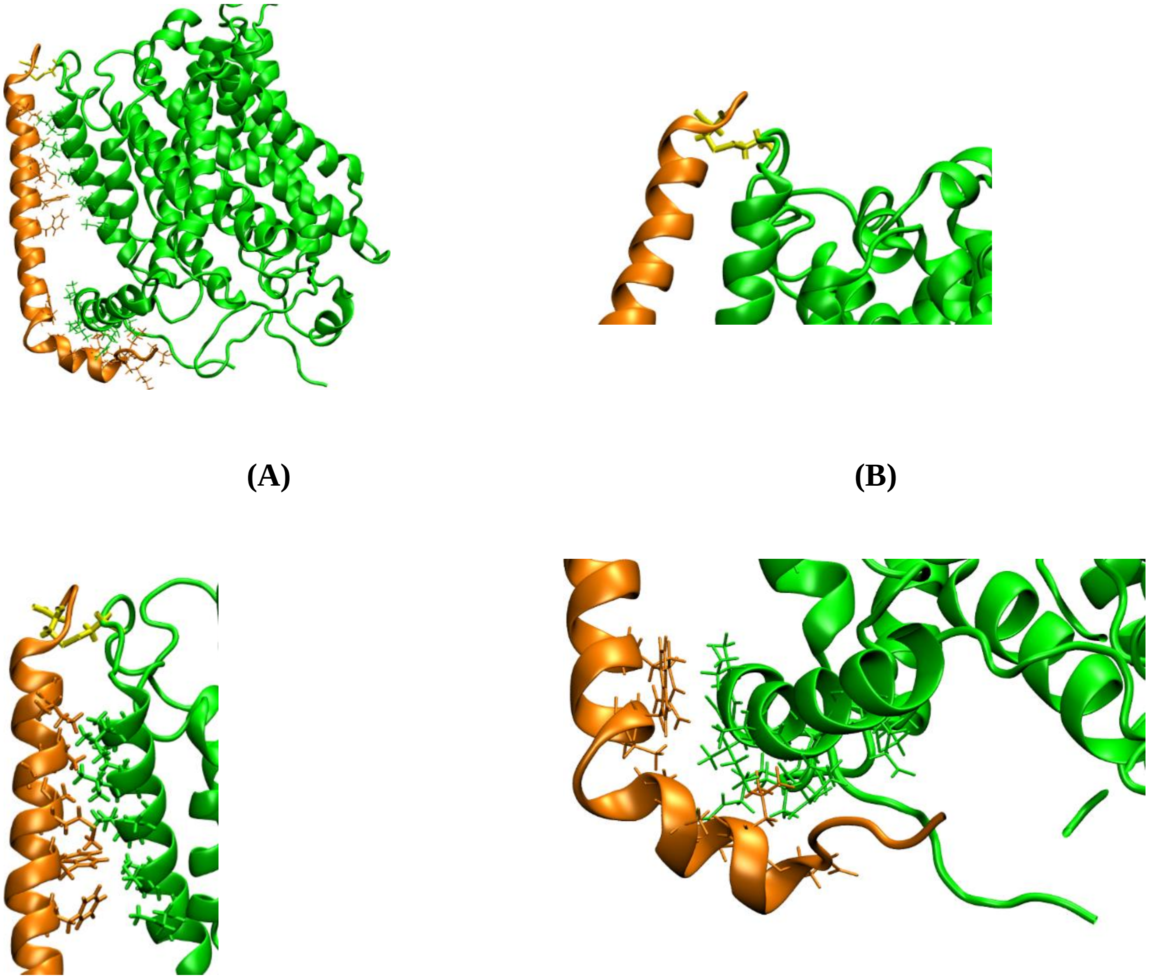

(A) (B)

(C) (D)

Figure S4. Interaction between 4F2hc and xCT

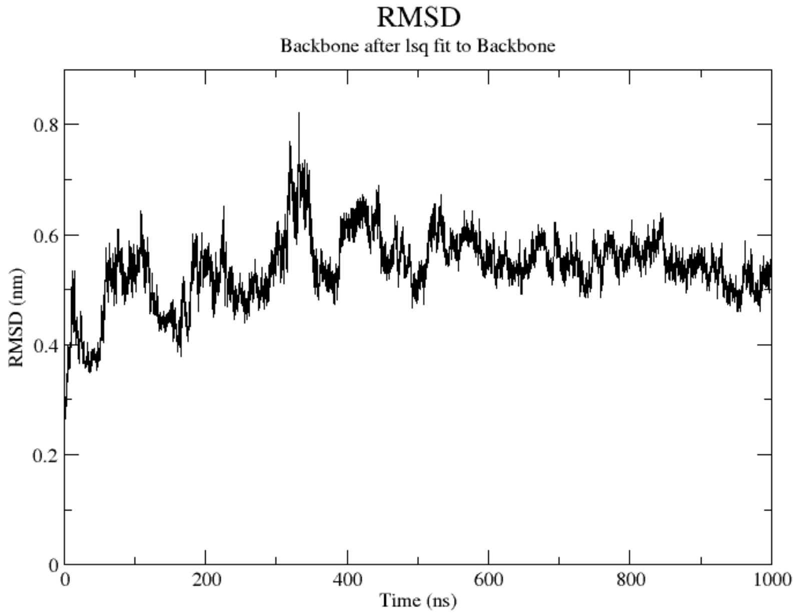

a

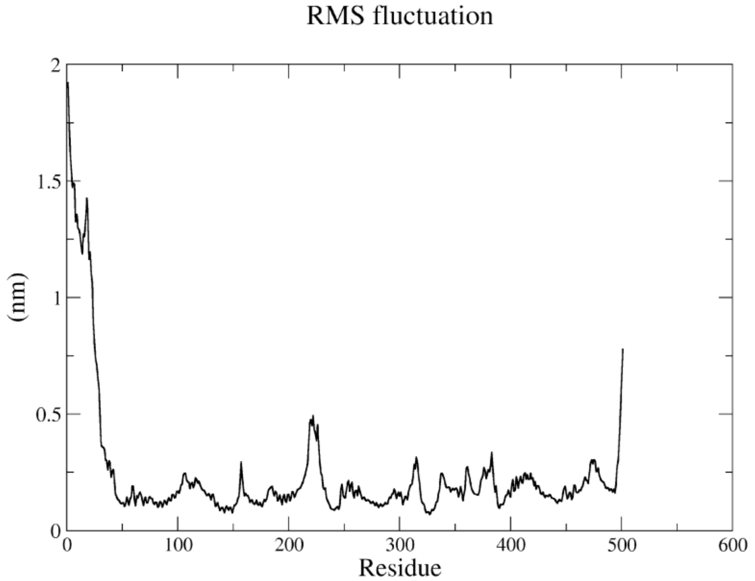

b

Figure S5. MD simulation analysis of inward-facing occluded conformation of xCT. **a)** Backbone RMSD (in nm) of model **283** during 1000 ns. **b)** Root-Mean-Square fluctuation (RMSF) for all residues of the light chain xCT during 500 ns simulation.

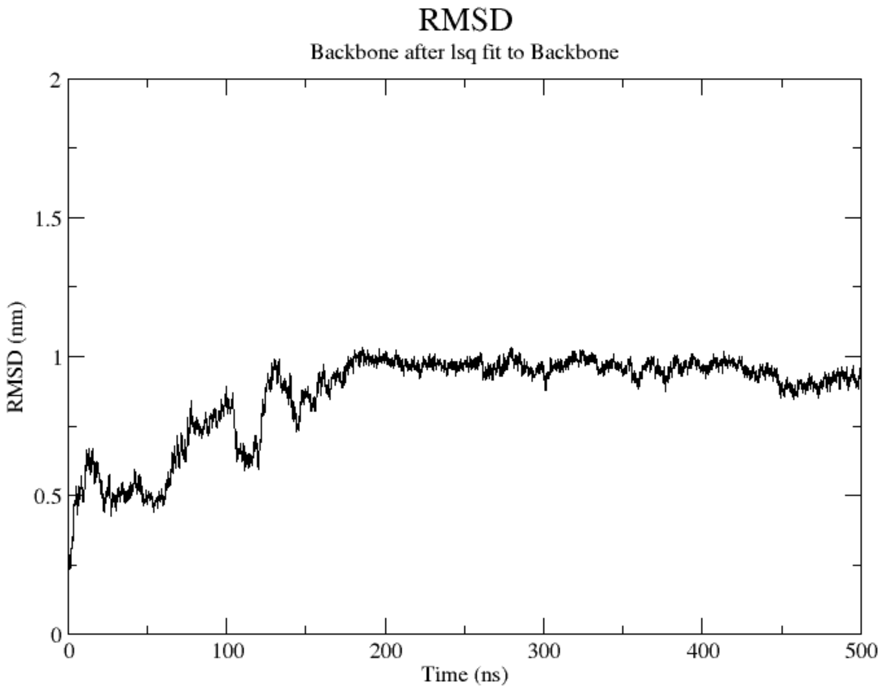

Figure S6. Backbone RMSD (in nm) of model **140** during 1000 ns.

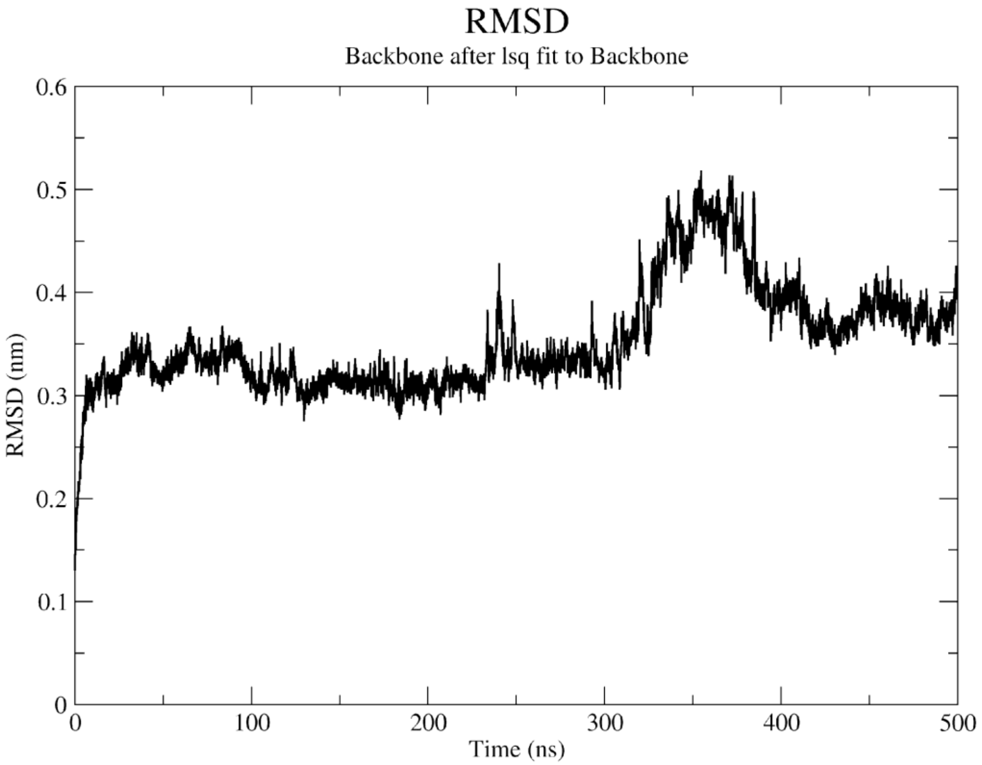

Figure S7. MD simulation analysis of outward open conformation of Xct

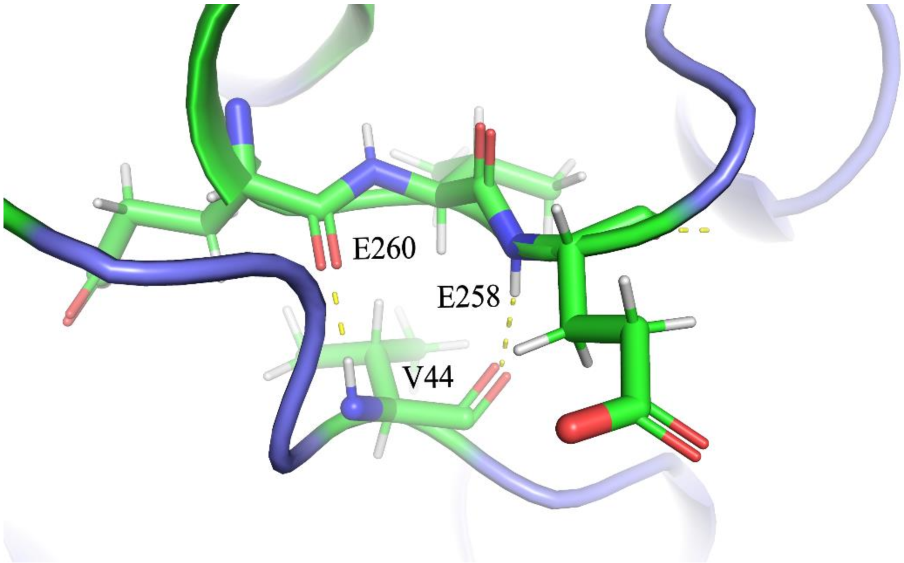

Figure S8. A close-up view of the interaction of TM6B and N-terminal helix (in outward open conformation)

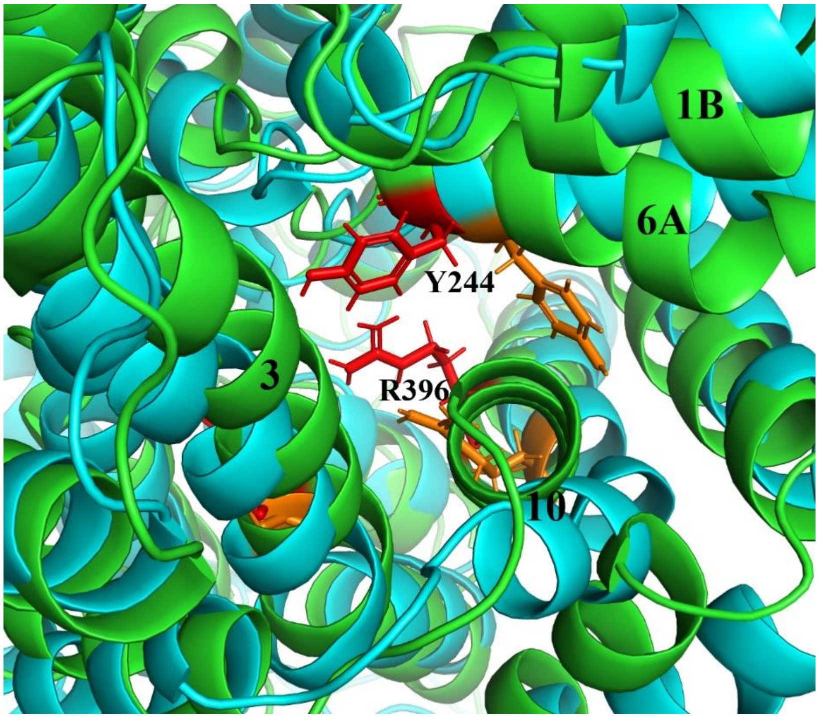

Figure S9. A close-up view of the geometries of R396 and Y244 residues in inward-open (green) and outward-open conformation (cyan).
